# Social Support, Perceived Stress, Socio-Demographic Factors and Relationship Quality among Polish Mothers of Prematurely Born Children

**DOI:** 10.3390/ijerph17113876

**Published:** 2020-05-30

**Authors:** Karolina Lutkiewicz

**Affiliations:** Institute of Psychology, University of Gdansk, 80-557 Gdansk, Poland; karolina.lutkiewicz@ug.edu.pl

**Keywords:** social support, stress, relationship, socio-demographic factors, premature birth

## Abstract

Families with a prematurely born child may be exposed to various difficulties associated with prematurity. The study aimed to explain the relationship between social support and the quality of the partnership among mothers of children born prematurely. In addition, the coexistence of social support, perceived stress and social-demographical factors in the neonatal period was examined. The study group consisted of 260 mothers of preterm born children. Subjects completed The Socio-Demographic Questionnaire, The Social Support Sources Questionnaire (SSQ), The Dyadic Adjustment Scale (DAS) and The Perceived Stress Questionnaire (PSQ). In the second phase of the study, mothers completed The Dyadic Adjustment Scale (DAS). Person’s correlation coefficients were used to examine the variables and hierarchical linear regression analyses were performed to examine the relative contributions of social support on partnership relation quality. The study results showed that social support is positively related to partnership relationship quality among mothers of preterm born children. Social support measured in the neonatal period is not connected with relationship quality measured in the early childhood period. Social support is related with the father’s level of education and perceived stress corresponds with the level of parents’ education. The findings highlight the importance of expanding supportive resources for couples dealing with premature birth, especially among those with a lower level of education and lower financial status.

## 1. Introduction

Pregnancy is a life-changing experience that may influence the functioning of the family system. Parents need to adjust to their new roles, routines, expectations, and demands on individuals’ and couples’ time and energy. Pregnancy and the postpartum period are special times, where new parents need a lot of attention and support.

Pre-term delivery is one of the most common pregnancy-related complications/health problems experienced by pregnant women [1,2]. The experience of premature delivery can be significant in the process of transition to parenthood and can change the functioning of the family during the first years of a child’s life [3]. Due to possible complications experienced during pregnancy, preterm delivery is recognized in the literature as a stressful and traumatic event for parents and their newborn child [4,5,6,7]. Difficult medical conditions that can put the baby’s life at risk, such as low birth weight, or long hospitalization in the neonatal intensive care unit may also have long-term consequences. These impact not only on the quality of interactions with the newborn child, but also the quality of the parental relationship [8]. As the consequences of preterm delivery on a child are well explored in the literature, the role of parental relationship quality in the context of maternal well-being or a child’s development needs still more attention.

Several studies that have explored relationship quality suggest that a high-quality partnership is the foundation for the development of a child and a source of valuable support and protection against severe stress and the negative consequences of difficult events in the family system [9,10]. Satisfactory relationships also promote good feelings related to the role of parenthood, which in turn can translate into better care for the child with increased parental responsiveness to the child’s needs [11]. Some researchers indicate that relationship quality is closely associated with social support and it can directly impact maternal well-being [9]. These perspectives highlight the need to further discuss how social support relates to partner relationships and stressful situations such as preterm birth.

Evidence has shown that social support is particularly important for all high-risk pregnancies and other problems related to procreation [4,5,6,7,12]. According to Juczyński (2016) social support can directly affect the perception of stressful situations and mitigates the negative effects of stressful events [13]. Thus, if social support is properly adjusted to the needs of the mother it may improve maternal well-being, decreasing the level of experienced stress and other risk factors such as depression symptoms during pregnancy [14,15]. When considering the stress experienced by both parents after premature birth, some studies also suggest that social support reduces parental stress and occurs as a protective factor [16]. Parents of prematurely born children can experience these higher levels of stress [17] due to the increased risk of adverse consequences in various aspects of the development of premature babies [18]. This additional stress may negatively impact the development of the child, parental relationship and the family unit. That is why, in the context of preterm birth it is important to also consider social support and its impact on the quality of the parental relationship. Adequate social support can significantly lower parental stress, and thus positively influence the parents and the prematurely born child’s well-being.

Zelkowitz et al., (2008) indicate that social support is one of the main aspects of relationship satisfaction [19], especially in the context of experienced stressful events. It helps to cope with intense emotions, works as a buffer against the negative effects of stress on the partner relationship [20] and strengthens the conviction that the difficult situation, such as the negative aspects of preterm birth, can be overcome [21]. According to research conducted by Khan and Aftah (2013) perceived social support is also a predictor of satisfaction within a partner relationship [22]. In addition, Lawrence et al. (2008) present social support as one of the main predictors affecting the relationship quality in the perinatal situation [23]. Other research demonstrates that support received from a partner also plays an important role in the process of transition to the role of mother [24,25] and can influence subjective relationship satisfaction [26,27,28]. A couple’s vulnerability to experiencing stress and their need for support is also linked to their socio-demographical background [9]. The influence of factors such as maternal age, education level or financial situation on experienced stress and support is still not fully understood. Some studies suggest that socio-economic status determines experienced stress by women and those with higher education use more productive strategies to deal with stressful situations [15]. Other studies demonstrate that stress and depressive symptoms are more likely to occur among mothers >35 years of age [29]. In addition, low socioeconomic status, younger age, and low education level may also be associated with higher stress and depressive symptoms within two years of delivery [30].

While many studies address the relationships between social support and stress on relationship quality, little research involves the analysis of how stress and social support impacts the partnership relationship in the context of preterm delivery [9]. Karney i Bradbury (1995) presented the Vulnerability–Stress–Adaptation (VSA) model, which indicates that stressful circumstances and enduring individual vulnerabilities to stress influence the partner relationships. The presented model shows that couples with more vulnerability features, have more difficulty to adapt to new roles during pregnancy and after delivery and have a tendency to negative interactions in relationships and frequent conflicts. This in turn lowers their relationship quality and increases inability to provide support. Similarly, a couple experiencing a stressful situation, such as preterm birth, based on their vulnerabilities, also may be exposed to difficulties in maintaining their relationship quality [31]. In addition, a model presented by Hatton et al. (2010) indicates that social support affects the quality of partner relationships after birth [32]. Despite the available studies, there is need for more detailed conceptualizations and models regarding sociocultural factors, as social support and the partner relationship can have a direct impact on birth outcomes [9].

As an addition to the above research, this study aims to examine: (a) the relationship between social support and the quality of the partners’ relationship; (b) the relationship between social support in the neonatal period and the quality of the partners’ relationship in the early childhood stage; (c) the relationship between social support and perceived stress by mothers; (d) if socio-demographical factors (parental age, parental education level, material status) are related to social support and perceived stress among mothers with prematurely born children. This paper aims to highlight the importance of expanding social support such as supportive resources and interventions for couples to overcome the stress of premature labor. Paying more attention to parental relationships, i.e., helping couples with a premature baby build more support between themselves can positively impact the development of the child and family system over the long term.

Based on the presented literature the study hypotheses were that: (1) social support affects the quality of the partnership relation in mothers of preterm born children; (2) social support received in the neonatal period is connected with the partners’ relationship quality in the early childhood stage; (3) perceived stress assessed by mothers with a premature baby is related to the level of social support. In this study the following explanatory questions were asked: If socio-demographical factors (parental age, parental education level, material status) are connected to social support experienced by mothers? If socio-demographical factors (parental age, parental education level, material status) are connected to perceived stress among mothers with prematurely born children?

## 2. Research Materials and Methods

In this study data were collected from mothers of preterm born children at the Department of Obstetrics at the Medical University of Gdansk. Screening all new admissions daily-identified potential participants. A research assistant met with potential participants to explain the study, determine eligibility, and review the informed consent form within two to four days of their infant’s birth. After a short introduction, women were invited to complete: (1) The Socio-Demographic Questionnaire—authors’ own work; (2) The Social Support Sources Questionnaire (SSQ) [33]; (3) The Dyadic Adjustment Scale (DAS) [34,35]; and (4) The Perceived Stress Questionnaire (PSQ) [36]. Secondly, mothers were invited to complete the socio-demographic form and The Dyadic Adjustment Scale (DAS) in the early childhood stage (between 24 and 30 months). All respondents were informed about the objectives of the study, the confidentiality of the data and signed the consent forms. Participants were also informed about the possibility to discontinue their participation at any time of the study. Participants were women, at least 18 years of age after the delivery of a preterm born child. Mothers were excluded, if they made a personal decision to withdraw from the study and/or if they suffered from a diagnosed mental illness. As this study focused on partner relationships, only women who reported being in an intimate relationship with a partner were considered for inclusion (formal or informal relationship N = 260). The Ethics Board of the Institute of Psychology of the University of Gdansk, Poland, approved the study.

There were 260 subjects in this study. The majority of women were married (79.7%), completed post-secondary education (60.3%) and were primigravidae (63.3%). Most mothers also had a single pregnancy (75.4%) and 84.1% of the responders had no pregnancy complications. Almost two-thirds had a gestational age between the 34, 35 or 36th week of pregnancy (64.4%, range 24–36 week). In terms of the financial status, nearly half of the surveyed women (128 people, 49.6%) described their material status as good, every third (84 people, 32.6%) respondent considered their material status as average, and 17.3% (45 people) considered it very good. Only three people (1.2%) considered their status to be lower than average. The mean age of women was 30, SD = 5.22 (range 18–45). A summary of the most important socio-demographic variables is presented in Table 1.

In this study the following self-reported measures were used to assess the data. (1) The Socio-Demographic Questionnaire—own work includes variables as follows: maternal age, parental education, place of living, maternal marital status, subjective assessment of financial family situation, number of children in the household, number of pregnancies, gestational age and information about pregnancy complications. (2) The Social Support Questionnaire (SSQ) [33] contains 14 statements that include three dimensions: social support obtained, desired and an assessment of satisfaction with support received in reality. The scale includes 14 different sources of social support, e.g., family, friends, health care workers, church and community. The reliability of the Polish version of the questionnaire for each dimension is as appropriate: support desirable (0.85), support obtained (0.69), support assessment (0.88) [33]. The amount of support is evaluated on a 4-point scale from 1 (‘not at all’) to 4 (‘extremely’), thus a total score for each sub-scale could range from 14 to 56 points [33,37]. In the SSQ questionnaire the Cronbach’s alpha reliability coefficient was 0.864. (3) The Dyadic Adjustment Scale (DAS) [34,35] uses a self-report method, consisting of 32 items that measure the relationship quality. The DAS includes four components of marital quality: dyadic consensus, dyadic cohesion, affectional expression and dyadic satisfaction [35]. The sum of the results in each category makes up the overall score. The total score can range from 0–151. A higher rating indicates a higher level of relationship quality. The Cronbach’s alpha reliability coefficient in the DAS scale equaled 0.96 and 0.73–0.94 for each subscale [38]. (4) The Perceived Stress Questionnaire (PSQ) is a 30-item questionnaire that assess the level of perceived stress from the last month [36]. The PSQ has seven dimensions: sense of fatigue, irritability, worrying, mental tension, lack of joy of life, sense of pressure and sense of being overwhelmed [39]. The sum of all obtained scores on each subscale can indicate severe stress. The higher the ratings the higher the level of perceived stress [40]. For the PSQ questionnaire the Cronbach’s alpha reliability coefficient equaled >0.9 [40].

In this study, to obtain the results statistical analysis was performed using the Statistical Package for Social Science (SPSS) version 24. Descriptive statistics were calculated. First, initial associations between the variables used in the study were examined through Persons’ correlations. In the next step linear hierarchical regression was calculated to examine the relative contributions of social support (predictor) and relationship quality (explained variable).

## 3. Results

### 3.1. Neonatal Period

Firstly, hypothesis 1 was examined to check if social support correlates with the quality of the partnership relation. The correlation analysis demonstrated that the variables were associated in the neonatal stage of the study. Results indicated that received support by mothers and the quality of that support is positively related with dyadic consensus, dyadic satisfaction and subjective assessment of the quality of the partnership of mothers of premature babies. These correlations were weak (0.14–0.16), with a significance level of *p* < 0.05 (Table 2).

### 3.2. Regression

In the next step, hierarchical regression analyses were performed to examine the relationship between social support and the partnership relation quality. In this analysis hypothesis 1 was verified, where the quality of the relationship was the explained variable. The results showed that received social support by mothers is significantly associated with their subjective judgment of partnership relation quality in two dimensions: dyadic consensus and dyadic satisfaction (Table 3).

### 3.3. Early Childhood Period

In the next step hypothesis 2 was verified. The correlation analysis showed that social support measured after delivery and the partnership’s quality measured in early childhood were not related with each other at a statistically significant level (Table 4). Hypothesis 2 was not confirmed.

### 3.4. Social Support and Perceived Stress

In order to investigate hypothesis 3, whether social support is related with mothers’ perceived stress, Pearson’s linear correlation was performed. The results showed that association between social support and perceived stress is statistically significant. The correlation is weak and exists in the following dimensions: irritability, lack of joy in life, pressure sensation and mental tension (Table 5).

### 3.5. Coexistence of Socio-Demographic Factors, Social Support and Perceived Stress

In further analysis it was investigated if socio-demographical factors are related with psychological variables. The correlation analyses were performed on selected socio-demographic factors (mother’s age, mother’s and father’s level of education, financial status) with perceived stress and social support measured in the neonatal period.

In terms of social support, it was shown that the received social support and the quality of this support correlated positively with the father’s education level. The correlations were mild, but statistically significant.

Analysis including perceived stress correlated negatively with the parental level of education (mother’s education and father’s education). Correlations were weak, but statistically significant. All stress derivatives (not including overload), i.e., feelings of exhaustion, irritability, worrying, psychological tension, lack of joy, feelings of pressure correlated negatively and were statistically significant with the education level of both parents. What is more, the results showed that worrying and feelings of pressure correlated negatively with the material status of the mother. The parental age is positively correlated with a lack of joy of life. A summary of the results is presented in Table 6.

## 4. Discussion

Many authors emphasize the role of social support as a crucial factor in the context of pregnancy and the postnatal period [4,5,6,7], but also in the case of high-risk pregnancy and any problems related to procreation [1,5,12]. The model proposed by Hatton and colleagues (2010) indicates support as one of the key factors that affects the quality of a relationship after the birth of a child [32,41]. Research conducted by Zelkowitz et al. (2008) [19] also considers support as the most significant aspect of the partner relationship, as it determines the development and quality of the partner relationship [41,42] and the satisfaction within it [32,43]. Similar relationships were obtained in this study. It has been shown that social support is a predictor of two dimensions in the quality of a partnership: consensus in relationships and satisfaction with relationships in mothers of premature babies in the neonatal period. Rostami, Ghazinour and Richter (2013) [27] have proven that support, especially received from a partner, is an important indicator of relationship satisfaction among women [23,26]. Based on their research, the authors claim that coherence in the relationship and emotional bond between the partners becomes stronger among couples, in which the partner is the main source of support. This in turn, leads to a higher assessment of the quality of the partner’s relationship by the partners. This study’s research results on social support in premature mothers corresponds with other research results. They indicate that the more support received, the higher the level of: quality of this support, consensus in the relationship, satisfaction in the relationship and quality of the partnership. These conclusions are consistent with the results of many other researchers who indicate that support contributes to a shared sense of happiness and satisfaction in marriage [28,44,45].

The obtained results did not show any significant correlations between social support received after delivery and the quality of the partnership in mothers of children born prematurely in the second stage. This study found that the level of social support in a neonatal relationship does not determine the further quality of the partnership in the group of mothers with premature babies.

### 4.1. Social Support and Perceived Stress

Numerous studies emphasize that social support is important in the context of experienced stressful situations in a relationship [23,28,46]. Social support is a buffer against the consequences of traumatic and stressful events [28,33,47]. The results of this study indicate that social support coexists with a sense of stress in mothers of premature babies in the neonatal period. The received support and the quality of this support in the subjective assessment of mothers of children born prematurely in the neonatal period is associated with stress and its elements such as: a sense of irritability, mental tension, the feeling of pressure and a greater joy of life. According to the research, received support, especially from the closest person, provides the psychological resources necessary to cope with stress, counteract difficult situations [28,48,49] and buffer the negative effects of stressful experiences [20]. In general, social support helps in maintaining good mental and physical health [28]. Research shows that a higher risk of depression occurs among women who receive less support than anticipated [26,47]. Social support is important among mothers of premature babies [50] and babies born with low birth weight as they are more vulnerable to stress, anxiety and depressive symptoms.

### 4.2. Socio-Demographical Factors, Social Support and Stress

In terms of social support, the presented research results suggest that the support received by the mother and the subjective assessment of the quality of this support are related to the father’s level of education. The mother receives more support of higher quality when the child’s father has a higher education. Mercer and Ferketich (1990) show that 90% of the support provided to the child’s mother comes from the father, which suggests that it is the child’s father who may play the primary role in providing social support to the mother [24,48]. The mother’s experiences in the perinatal period should be considered individually [51]. In terms of social support, it is important to adjust the level of support to the needs of the child’s mother, which primarily reduces the level of experienced stress [15].

The results of this study show that perceived stress corresponds with the level of parents’ education. In addition, all derivatives of stress in mothers of premature babies, i.e., fatigue, irritability, worry, mental tension, lack of joy, and pressure are associated with the education of both parents. Some available research in this area suggests that younger, less educated individuals with lower income tend to experience greater difficulties when becoming a parent [52]. Kurdek (1993) claims that this trend mainly concerns the female population [53] On the other hand, other studies indicate that the birth of a child is more difficult for people with high economic status, especially mothers who have given up high status and high earnings to devote themselves to the role of mother [54]. Studies by Schappin et al. (2013), suggest that a mother’s education is not associated with parental stress levels. According to the authors, mothers with higher education experience less stress, as they have better coping skills with difficulties compared to those with lower levels of education [15]. The obtained results also suggest that the feeling of pressure and worrying by premature mothers is related to the material status of the mother. The obtained results correspond to the results from the research of Schappin et al. (2013), according to which, low socioeconomic status can contribute to an increase in the level of stress experienced in a partner relationship [15].

Studies indicate that a mother’s age plays an important role in experiencing parental stress. This study has shown that the older the parents of the child, the less joy of life they experience. Some research demonstrated that depression is significantly higher among women who gave birth between 35 and 44 years of age than women in younger age groups [29]. Mayberry, Horovitz and Declercq (2007) proved that young mothers, with low income, lower level of education and more children, more often reported depressive symptoms within two years of delivery [30]. Contrary research made by Schappin et al. (2013) [15], showed that mothers around the age of 30, when compared to a group of women who became mothers in older age, had a more stable life and stable network of social relations. Therefore, even in difficult situations, such as the prolonged need for their child to be in hospital after delivery, they experienced stress at a lower level. Young age (<18) [55] and old age (>35) [1,49] could be risk factors for postpartum depression in pregnant women.

Nevertheless, the coexistence of social factors with support and a sense of stress are ambiguous and still limited. Studies show that women with premature babies do not create homogeneous groups and each case should be considered individually. These may be impacted by a baby’s characteristics, birth weight, gestational age, and a mother’s individual experience of prematurity and socio-demographical background [51].

## 5. Limitations

This study includes some methodological shortcomings. In this prospective study, the quality of the partner relationship was examined only in the mothers’ group. Including fathers in the study would show the role of social support, perceived stress and partner quality in a broader perspective, helping to understand how premature birth affects the functioning of men in the relationship and general functioning of the couple.

Pregnancy complications can be a greater burden for mothers and couples. In the study, most of the subjects (84.1%) had a healthy pregnancy. Attention should be paid to pregnancy complications, which can be a greater burden for mothers and couples. It would be interesting to take under consideration social support among mothers of infants with, for example, very low birth weight or extremely premature infants who are exposed to more invasive treatments associated with all morbidities [56]. Based on gestational age, the sample in this study could appear as a limitation. More than half the women gave birth at 34, 35 or 36 weeks, which is considered late prematurity. The sample was composed of Polish mothers who were predominantly well educated (60.3% higher education); replications of our results with a more heterogeneous sample would foster generalization of findings to a broader population. Taking under consideration socio-demographical factors on the quality of the partner’s relationship among mothers would also broaden the perspective on a couple’s relationship after the birth of a preterm baby.

In further research it would be valuable to gather additional information about specific sources of social support. This would help understand what resources can be improved for parents dealing with prematurity. Another limitation of the study includes its methods, as all questionnaires were self-reports. The presented results should also be interpreted with a high degree of caution, because although the correlations were statically significant, they were weak. Additionally, it does not present the causality between measured variables, so the interpretation of the results is limited.

## 6. Conclusions

The presented study concentrates on the relationship quality, social support and experienced stress by mothers with prematurely born babies. The findings emphasize the couples’ relationship and the link between social support and stress among mothers, also including socio-demographical factors. In the case of premature birth, social support should be the overriding element of psychological help for both parents. The focus should include mothers and couples with a lower level of education and lower material status. Programs in hospitals and community resources should pay more attention to parental relationships, e.g., how may a couple with a premature baby receive more social support from different resources and how can the couple build more support between themselves. These actions may reduce stress in the mothers of premature babies and could improve the child’s development [57]. Resources may also include interventions, which may help increase the long-term commitment of the father to parenting and maternal support [41].

## Figures and Tables

**Table 1:** Socio-demographic variables of the study group.

Variable	*M* (*SD*)/Med	%	Minimum	Maximum
Maternal age	30.00 (5.22)	–	18.0	45.0
Maternal education	–	60.3% post-secondary	–	–
Place of living	–	75.9% city	–	–
Number of children	–	62.8% no children	0	4
Financial status	–	49.6% good	–	–
Paternal age	32.07 (5.78)	–	17.0	51.0
Paternal education	–	48.3% higher education	–	–
Gestational age	34.0	–	24	36

M—Mean, Med—Median, SD—Standard Deviation.

**Table 2:** Pearson’s linear correlation coefficient for a relationship satisfaction and its components with social support.

Variable	M (SD)	Desired Support	Received Support	Quality of Received Support
Consensus	52.78 (7.24)	0.03	0.16 *	0.16 *
Cohesion	19.53 (3.68)	0.04	0.06	0.04
Satisfaction	41.32 (5.25)	0.04	0.14 *	0.14 *
Affectional expression	10.42 (1.74)	−0.06	0.06	0.04
General satisfaction	124.05 (15.09)	0.03	0.15 *	0.14 *

M—Median, SD—Standard Deviation, * *p <* 0.05; ** *p <* 0.01.

**Table 3:** Linear hierarchical regression analysis, in which the quality of the partnership and its components were explained variables.

	Consensus		Cohesion		Satisfaction		Affectional Expression		General Satisfaction	
	*β*	Δ*R*^2^	*β*	Δ*R*^2^	*β*	Δ*R*^2^	*β*	Δ*R*^2^	*β*	Δ*R*^2^
**Desired support**	−0.07		0.001		−0.11		−0.15		−0.09	
**Received support**	0.17 *		−0.02		−0.17 *		0.14		−0.15 *	
**Total** ***R*** **^2^**		0.563 **		0.312 **		0.487 **		0.336 **		0.595 **

* *p <* 0.05; ** *p <* 0.01.

**Table 4:** Pearson’s linear correlation coefficient for social support and a general relationship satisfaction and its components in the second stage of the research.

Variable	*M* (*SD*)	Desired Support	Received Support	Quality of Received Support
Consensus-2	49.73 (7.69)	0.27	0.23	0.09
Cohesion-2	18.11 (5.14)	0.02	0.09	0.04
Satisfaction-2	36.54 (5.91)	0.03	0.17	0.10
Affection expression-2	9.14 (1.83)	0.26	−0.13	−0.23
General satisfacion-2	113.51 (17.64)	0.16	0.17	0.06

Annotation: M—Median. SD—Standard Deviation * *p <* 0.05; ** *p <* 0.01.

**Table 5:** Pearson’s linear correlation coefficient for social support and mother’s perceived stress and its components.

Variable	*M* (*SD*)	RS	QRS	DS	PS	I	W	MT	L	FP	O
Desired Support	37.61 (7.04)	0.61 **	0.49 **	0.06	−0.02	−0.14 *	0.08	0.00	−0.001	0.03	0.07
Received Support	33.62 (5.41)		0.87 **	−0.09	−0.07	−0.20 **	−0.06	−0.10	−0.15 *	−0.15 *	−0.01
Quality of Received Support	34.26 (6.01)			−0.10	−0.08	−0.21 **	−0.11	−0.14 *	−0.17 *	−0.17 *	−0.03
Perceived Stress	56.47 (17.1)				0.84 **	0.56 **	0.86 **	0.79 **	0.90 **	0.71 **	0.75 **
Sense of Exhaustion	9.23 (2.24)					0.61 **	0.75 **	0.71 **	0.72 **	0.55 **	0.58 **
Irritability	4.37 (1.25)						0.56 **	0.63 **	0.56 **	0.51 **	0.42 **
Worrying	9.19 (2.92)							0.79 **	0.73 **	0.65 **	0.67 **
Mental Tension	6.91 (2.36)								0.70 **	0.64 **	0.60 **
Lack of joy	13.85 (3.78)									0.64 **	0.62 **
Feeling of Pressure	6.04 (2.08)										0.66 **
Overload	7.97 (2.63)										

Annotation: DS—Desired Support, RS—Received Support, QRS—Quality of the Received Support, PS—Perceived stress, SE—Sense of Exhaustion, I—Irritability, W—Worrying, MT—Mental Tension, L—Lack of joy of life, FP—Feeling of Pressure, O—Overload. * *p <* 0.05; ** *p <* 0.01.

**Table 6:** Pearson’s linear correlation coefficients and point-two-series correlation coefficients for the relationship of psychological variables with social variables.

Variable	*M* (*SD*)	Maternal Age ^a^	Paternal Age ^a^	Maternal Education ^b^	Paternal Education ^b^	Marital Status ^b^	Financial Status ^b^
Desired Support	37.61 (7.04)	−0.03	−0.09	0.10	0.11	0.07	0.01
Received Support	33.62 (5.41)	−0.02	−0.04	0.08	0.18 *	0.09	0.08
Quality of Received Support	34.26 (6.01)	0.02	0.01	0.14	0.22 **	0.12	0.07
Perceived Stress	56.47 (17.1)	0.12	0.09	−0.24 **	−0.27 **	−0.04	−0.08
Sense of Exhaustion	9.23 (2.24)	0.12	0.07	−0.15 *	−0.18 *	−0.07	−0.03
Irritability	4.37 (1.25)	−0.11	−0.08	−0.23 **	−0.27 **	−0.13	−0.12
Worrying	9.19 (2.92)	0.05	0.01	−0.26 **	−0.29 **	−0.07	−0.17 *
Mental Tension	6.91 (2.36)	0.01	−0.05	−0.19 *	−0.24 **	−0.04	−0.07
Lack of joy	13.85 (3.78)	0.15 *	0.15 *	−0.27 **	−0.29 **	−0.02	−0.12
Feeling Pressure	6.04 (2.08)	0.10	0.05	−0.22 **	−0.28 **	−0.11	−0.24 **
Overload	7.97 (2.63)	0.08	0.06	−0.13	−0.12	−0.001	−0.10

Annotation: Maternal Education (0—primary, vocational, secondary, 1—higher education), Paternal Education, Marital Status (0—informal partnership, 1—marriage), Financial Status (0—lower than average, average, 1—good, very good), * *p* < 0.05; ** *p* < 0.01. ^a^ Pearson’s linear correlation coefficients; ^b^ Point-two-series correlation coefficients.
